# High Sensitivity of SIRT3 Deficient Hearts to Ischemia-Reperfusion Is Associated with Mitochondrial Abnormalities

**DOI:** 10.3389/fphar.2017.00275

**Published:** 2017-05-16

**Authors:** Rebecca M. Parodi-Rullán, Xavier Chapa-Dubocq, Pedro J. Rullán, Sehwan Jang, Sabzali Javadov

**Affiliations:** Department of Physiology, University of Puerto Rico School of Medicine, San JuanPR, United States

**Keywords:** heart, mitochondria, SIRT3, ischemia-reperfusion, sanglifehrin A, protein acetylation

## Abstract

**Aim:** Sirtuins are NAD^+^-dependent deacetylases that regulate cell metabolism through protein acetylation/deacetylation, and SIRT3 is the major deacetylase among mitochondrial isoforms. Here, we elucidated the possible role of acetylation of cyclophilin D, a key regulator of the mitochondrial permeability transition pore (mPTP), in mitochondria-mediated cardiac dysfunction induced by ischemia-reperfusion (IR) in wild type (WT) and SIRT3 knockout (SIRT3^-/-^) mice.

**Materials and Methods:** Isolated and Langendorff-mode perfused hearts of WT and SIRT3^-/-^ mice were subjected to 25-min global ischemia followed by 60-min of reperfusion in the presence or absence of the mPTP inhibitor, sanglifehrin A (SfA).

**Results:** Analysis of mitochondrial sirtuins demonstrated that SIRT3 deficiency upregulated SIRT4 with no effect on SIRT5 expression. Hearts of SIRT3^-/-^ mice exhibited significantly less recovery of cardiac function at the end of IR compared to WT mice. Intact (non-perfused) SIRT3^-/-^ hearts exhibited an increased rate of Ca^2+^-induced swelling in mitochondria as an indicator of mPTP opening. However, there was no difference in mPTP opening and cyclophilin D acetylation between WT and SIRT3^-/-^ hearts subjected to IR injury. Ca^2+^-stimulated H_2_O_2_ production was significantly higher in SIRT3^-/-^ mitochondria that was prevented by SfA. Superoxide dismutase activity was lower in SIRT3^-/-^ heart mitochondria subjected to IR which correlated with an increase in protein carbonylation. However, mitochondrial DNA integrity was not affected in SIRT3^-/-^ hearts after IR.

**Conclusion:** SIRT3 deficiency exacerbates cardiac dysfunction during post-ischemic recovery, and increases mPTP opening and ROS generation without oxidative damage to mitochondrial proteins and DNA.

## Introduction

Amongst all post-translational modifications, acetylation is thought to be the most common and important for mitochondrial proteins. Over 60% of mitochondrial proteins contain acetylation sites and most of them are involved in energy metabolism (Baeza et al., 2016). Protein acetylation, at least in the mitochondria, has an inhibitory role, and consequently, an acetylated mitochondrial proteome would result in inhibition of mitochondrial metabolism and ATP synthesis. Acetylation/deacetylation of mitochondrial proteins is regulated by sirtuins. Sirtuins are NAD^+^-dependent deacetylases that play an essential role in cell metabolism as energy and redox sensors (Kim et al., 2006). Mammals contain seven sirtuin isoforms (SIRT1–7), three (SIRT3–5) of which are localized in the mitochondria. SIRT3 is the major mitochondrial deacetylase (Lombard et al., 2007) that deacetylates and stimulates the activity of metabolic enzymes involved in fatty acid oxidation (Hirschey et al., 2010; Bharathi et al., 2013), glucose oxidation (Ozden et al., 2014), tricarboxylic acid cycle (Yu et al., 2012), and electron transfer chain (ETC) complexes (Vassilopoulos et al., 2014). The contribution of protein acetylation to coronary heart diseases such as IR has not been elucidated.

Mitochondria play a crucial role in mediating oxidative stress signaling during cardiac IR by regulating cell death through apoptosis and necrosis, depending on ATP levels in cardiomyocytes. High levels of Ca^2+^ and ROS in the heart during cardiac IR have been shown to induce mitochondrial permeability transition (mPT) leading to opening of the mPT pores (mPTP) in the inner mitochondrial membrane (Crompton and Costi, 1988; Griffiths and Halestrap, 1995). Despite many studies, the molecular identity of the mPTP complex remains unclear. It was initially thought that mPTP was composed of the adenine nucleotide translocator and voltage-dependent anion channel. However, genetic studies revealed that these proteins are dispensable for mPTP formation (Kokoszka et al., 2004; Baines et al., 2007). Likewise, studies on phosphate carrier (P_i_C) KO animals/cells found that P_i_C deficient mitochondria are still prone to mPTP induction (Gutierrez-Aguilar et al., 2014). Most recent studies focus on the F_0_F_1_-ATP synthase as a core mPTP component (Giorgio et al., 2013; Alavian et al., 2014) although many questions concerning its potential role remain unanswered. Notably, CyP-D, a matrix-located *cis*-*trans*-isomerase, is the only protein broadly accepted as a major regulator of pore formation. Genetic ablation or pharmacological inhibition of CyP-D increased the resistance of mitochondria to Ca^2+^-induced swelling as a marker of mPTP opening (Javadov et al., 2003; Baines et al., 2005; Basso et al., 2005). Theoretically, activation of CyP-D and its interaction with a target protein(s) to form the mPTP pore can be mediated through several mechanisms (Reviewed in Javadov et al., 2017).

CyP-D has been shown to undergo post-translational modifications through phosphorylation (Rasola et al., 2010), nitrosylation (Kohr et al., 2011), and acetylation (Hafner et al., 2010; Shulga and Pastorino, 2010). Previous studies revealed a link between CyP-D acetylation and cardiac dysfunction. We previously demonstrated that post-infarction heart failure in rats induced downregulation of SIRT3 and increased CyP-D acetylation and mPTP opening (Parodi-Rullan et al., 2012). CyP-D was acetylated on lysine 166 (K166), and acetyl-CyP-D was shown to be deacetylated by SIRT3. In addition, SIRT3^-/-^ mice developed age- and stress-induced cardiac hypertrophy (Hafner et al., 2010). Attenuation of cardiac hypertrophy by SIRT3 was associated with deacetylation and nuclear translocation of FoxO3 whereby it stimulated transcription of antioxidant enzymes (Sundaresan et al., 2009). Acetylation of mitochondrial SOD2 reduced its activity, and SIRT3-induced deacetylation recovered the activity enhancing the antioxidant capacity of the mitochondria (Tao et al., 2010). Importantly, previous studies on SIRT3 were conducted mostly on non-cardiac cells that limit the understanding of the contribution of SIRT3 to mitochondrial function in the heart under both physiological and pathologic conditions.

In this study, we investigated the role of SIRT3 in maintaining mitochondrial function using WT and SIRT3^-/-^ mice. Hearts isolated from WT and SIRT3^-/-^ mice were exposed to global IR using a Langendorff-mode perfusion to assess the effects of SIRT3 ablation on post-ischemic recovery, ROS production, and mPTP susceptibility. Results demonstrated that SIRT3 ablation does not induce protein oxidation and mtDNA damage in intact heart mitochondria presumably due to activation of compensatory mechanisms. On the other hand, intact mitochondria from SIRT3 deficient hearts were more susceptible to Ca^2+^-induced mPTP formation. Similarly, exposure of SIRT3^-/-^ hearts to IR diminishes post-ischemic recovery of cardiac function associated with increased protein oxidation and ROS production.

## Materials and Methods

### Animals

Three-month-old male adult WT (129S1/SvImJ) and SIRT3^-/-^ (Sirt3^tm1.1Fwa^) mice (20–25 g) were purchased from Jackson Laboratory (Bar Harbor, ME, United States). All experiments were performed according to protocols approved by the UPR Medical Sciences Campus Animal Care and Use Committee and conformed to the National Research Council Guide for the Care and Use of Laboratory Animals published by the US National Institutes of Health (2011, eighth edition).

### *Ex Vivo* Model of IR

Hearts isolated from WT and SIRT3^-/-^ mice were evaluated in the following six groups: (1) WT, WT hearts (not perfused); (2) S3 SIRT3^-/-^ (not perfused); WT-IR, WT hearts subjected to IR; WT-IS, WT hearts subjected to IR in the presence of 0.2 μM SfA (mPTP inhibitor); S3-IR, SIRT3^-/-^ hearts subjected to IR; S3-IS, SIRT3^-/-^ hearts subjected to IR in the presence of 0.2 μM SfA. Mice were anesthetized using tribromoethanol (Avertin^®^) anesthesia at a dose of 250 mg/kg, IP. Once anesthetized, the animal was heparinized and the chest cavity opened to allow exposure of the heart. To induce IR, the aorta was identified, cut, and cannulated *in vivo*. The heart was then rapidly excised and the cannula connected to the Langendorff perfusion setup. The heart was perfused as previously described (Porter et al., 2014), at a constant flow of 4 ml/min with Krebs-Henseleit solution containing: 1.2 mM KH_2_PO_4_, 1.2 mM MgSO_4_, 2.5 mM CaCl_2_, 4.7 mM KCl, 118 mM NaCl, 25 mM NaHCO_3_, and 10 mM glucose equilibrated at 95% O_2_ and 5% CO_2_, pH 7.4 at 37°C. Left ventricular pressures were recorded by insertion of a water-filled balloon connected to a pressure transducer in the left ventricle. Functional parameters with continuous monitoring included: heart rate (HR), left ventricular systolic (LVSP), and end diastolic (LVEDP) pressure. LVDP was calculated as the difference between LVSP and LVEDP (LVDP = LVSP–LVEDP). The rate-pressure product (RPP) was used to estimate cardiac work and calculated as the product of LVDP and HR (RPP = LVDP^∗^HR). Labscribe2 Data Acquisition Software (iWorx 308T, Dover, NH, United States) was used for pressure recordings. Global normothermic ischemia was induced by stopping the perfusion for 25 min with the heart immersed in deoxygenated Krebs–Henseleit solution at a constant temperature of 37°C. Reperfusion was allowed for 1 h by restoring the same pre-ischemic flow rate for the heart. SfA was added 10 min prior to ischemia and present throughout ischemia and reperfusion.

Samples of the coronary effluent were collected prior to ischemia and during reperfusion for analysis of the lactate dehydrogenase (LDH) activity. The activity of LDH in the coronary effluent was measured spectrophotometrically at 340 nm at different time points, as previously described (Jang and Javadov, 2014) with minor modifications.

### Mitochondria Isolation

#### Isolation of Cardiac Mitochondria

Heart ventricles were cut and incubated in 0.05% Trypsin-EDTA for 10 min and then, were homogenized using a Polytron homogenizer in 2 ml of ice-cold sucrose buffer containing: 300 mM sucrose, 20 mM Tris-HCl, and 2 mM EGTA and supplemented with 0.05% BSA. Homogenate was then centrifuged at 700 ×*g* for 10 min, to remove cell debris. Supernatant was centrifuged at 7,500 ×*g* for 10 min to precipitate mitochondria. The final pellet was washed twice by centrifugation at 7,000 ×*g* for 10 min using sucrose buffer. Final pellet containing mitochondria was resuspended in 100 μl of sucrose buffer.

#### Isolation of Liver Mitochondria

In addition to heart, mitochondria were isolated from intact livers of WT and SIRT3^-/-^ mice to compare biochemical and genetic parameters between cardiac and liver mitochondria. Mouse liver was cut and homogenized using a Polytron homogenizer in 2 ml of ice-cold sucrose buffer containing: 300 mM sucrose, 20 mM Tris-HCl, and 2 mM EGTA. Homogenate was then centrifuged at 2,000 ×*g* for 3 min, to remove cell debris. Supernatant was then centrifuged at 10,000 ×*g* for 15 min to precipitate mitochondria. The final pellet was washed once with sucrose buffer by centrifugation at 10,000 ×*g* for 10 min. Mitochondria-enriched pellet was resuspended in 200 μl of sucrose buffer.

### mPTP Opening

Swelling of de-energized mitochondria as an indicator of mPTP opening in the presence or absence of Ca^2+^ was determined by monitoring the decrease in light scattering at 545 nm as described previously (Jang and Javadov, 2014).

### Total ROS Production

Mitochondria H_2_O_2_ production was determined as increased AmplexRed^®^(Molecular Probes, Eugene, OR, United States) fluorescence at excitation 530 nm and emission 560 nm.

### Enzymatic Activity of ETC Complexes

Mitochondrial samples were quantified and normalized to 0.1–0.3 μg/μl of mitochondrial protein in mitochondrial lyse buffer containing 2 mM EDTA and 0.1% Triton X-100. Normalized mitochondria were freeze-thawed two times before their use in enzymatic analysis to destroy mitochondrial membranes and provide access of substrates to ETC complexes. Mitochondrial complex activity was determined as previously described (Hernandez et al., 2014) with minor modifications. All assays were performed at the SpectraMax^®^M Series Multi-Mode Microplate Reader (Molecular Devices) at 37°C.

The activity of *citrate synthase* was determined spectrophotometrically by measuring coenzyme A formation at 412 nm as described previously (Parodi-Rullan et al., 2012).

### Total Antioxidant Capacity (TAC) and SOD Activity

The total antioxidant capacity (TAC) and SOD activity were determined in equal amounts of mitochondrial protein in accordance with manufacturer’s instructions using the TAC and SOD assay kits (Sigma–Aldrich). Briefly, TAC was measured as the reduction of Cu^2+^ and expressed in 6-hydroxy-2,5,7,8-tetramethylchroman-2-carboxylic acid (Trolox) equivalents. The activity of SOD was determined as percent inhibition of reduction of 2-(4-iodophenyl)-3-(4-nitrophenyl)-5-(2,4-disulfophenyl)-2H-tetrazolium.

### SDS-PAGE and Western Blotting

Equal amounts of homogenate or mitochondrial protein were resolved by SDS-PAGE and transferred onto Amersham Hybond ECL nitrocellulose membranes (GE Healthcare Bio-Sciences). The membranes were immunoblotted with acetylated lysine (Cell Signaling), SIRT3 (Cell Signaling), SIRT4 (Santa Cruz), SIRT5 (Santa Cruz), cytochrome *c* oxidase subunit IV (COXIV, Santa Cruz), OGG-1 (Abcam), or APE-1 (Abcam) antibodies followed by IRDye^®^(LI-COR Biosciences) secondary antibodies. Bands were visualized using ODYSSEY^®^CLx (LI-COR Biosciences) infrared scanner. The resulting images were analyzed with ImageJ (NIH).

### Co-immunoprecipitation

Immunoprecipitation experiments were performed following the recommended protocol of Dynabeads (Invitrogen-Life Technologies). Proteins containing acetylated lysine residues were immunoprecipitated from mouse heart homogenate or liver mitochondrial extracts using an antibody against acetylated lysine residues (Cell Signaling). The immunoprecipitates were separated by SDS-PAGE, blotted onto Amersham Hybond ECL nitrocellulose membranes (GE Healthcare Bio-Sciences) and the western blots developed using antibody against SOD2 (Santa Cruz), and CyP-D (Abcam) followed by secondary antibodies. Bands were visualized using the Molecular Imager VersaDoc^TM^ MP 4000 (Bio-Rad).

### Protein Carbonylation Assay

Protein carbonyls were analyzed as described previously (Escobales et al., 2014). Briefly, an aliquot of perfused or intact heart, or intact liver mitochondrial protein was derivatized with dinitrophenylhydrazine (Sigma–Aldrich) under acid denaturing conditions. Proteins were separated by SDS-PAGE and subjected to western blotting with anti-dinitrophenyl primary antibodies (Sigma–Aldrich) at 1:1000 dilution followed by by IRDye^®^(LI-COR Biosciences) secondary antibodies. Bands were visualized using ODYSSEY^®^CLx (LI-COR Biosciences) infrared scanner. In order to correct for non-specific binding of antibodies, separate aliquots of the mitochondrial proteins that had been acid-denatured but not treated with dinitrophenylhydrazine were run in parallel.

### Analysis of mtDNA Lesions

Total DNA was extracted from heart and liver tissue samples using the QIAGEN DNEASY Blood & Tissue Kit (QIAGEN), quantified using Quant-iT^TM^ PicoGreen^®^dsDNA Assay Kit (Invitrogen) following manufacturer’s instructions and normalized to 5 ng/μl. Mitochondrial short (2 kbp) and long (15 kbp) fragments were used to determine mtDNA damage (Barreto-Torres et al., 2015). Total DNA damage was determined in liver samples by quantifying 8-hydroxy-2′-deoxyguanosine (8-oxo-dG) levels using DNA/RNA Oxidative Damage EIA Kit (Cayman Chemical) following manufacturer’s instructions.

### Statistical Analysis

Data are presented as means ± SEM. Statistical significance was evaluated using Prism Graph Pad (San Diego, CA, United States) using two-way ANOVA followed by Tukey’s multiple comparison *post hoc* test, one-way ANOVA followed by Sidak’s multiple comparison *post hoc* test, or an unpaired 2-tailed Student’s *t*-test. Differences were considered to be statistically significant when *P* < 0.05.

## Results

### SIRT3^-/-^ Mice Demonstrate Low Recovery of Cardiac Function in Response to IR

**FIGURE 1:**
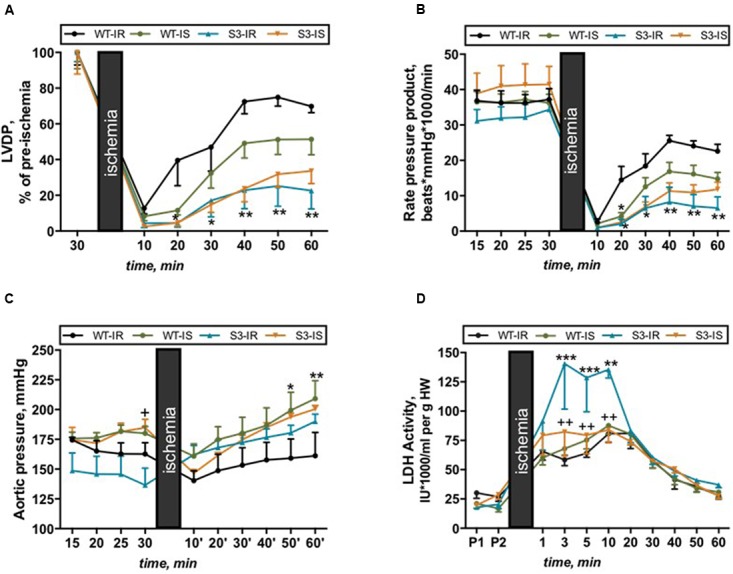
**Cardiac function (A–C)** and LDH release **(D)** in Langendorff-perfused hearts isolated from WT and SIRT3^-/-^ mice and subjected to IR in the presence or absence of SfA. **(A)** Left ventricle developed pressure (LVDP) expressed as percent recovery of its pre-ischemic values, **(B)** Cardiac work represented by the RPP, **(C)** Coronary pressure values shown in mmHg, and **(D)** LDH activity normalized to heart weight (HW). Groups: WT-IR, WT subjected to IR; WT-IS, WT subjected to IR in the presence of 0.2 μM SfA; S3-IR, SIRT3^-/-^ subjected to IR; S3-IS, SIRT3^-/-^ subjected to IR in the presence of 0.2 μM SfA. ^∗^*P <* 0.05, ^∗∗^*P <* 0.01, ^∗∗∗^*P <* 0.001 vs. WT-IR; ^+^*P <* 0.05, ^++^*P <* 0.01 vs. S3-IR. *n* = 5–7.

There was phenotypically no difference between intact (non-perfused) WT and SIRT3^-/-^ *hearts that demonstrated similar body* weight (BW), heart weight (HW), and HW/BW ratio (*data not shown*). In order to assess the effects of SIRT3 ablation on cardiac function, hearts of WT and SIRT3^-/-^ mice were subjected to *ex vivo* IR in the presence and absence of the mPTP inhibitor, SfA. We found that SIRT3 deficient hearts are more sensitive to global IR as they exhibited a 68 and 71% (*P* < 0.001 for both) less LVDP and RPP, respectively, by the end of reperfusion (**Figures 1A,B**, WT-IR vs. S3-IR). SfA (mPTP inhibitor) administered 10 min prior to ischemia and throughout reperfusion had no effect on post-ischemic recovery of both WT and SIRT3^-/-^ mice (**Figures 1A,B**).

Notably, SfA increased coronary pressure in WT hearts by 30% (*P* < 0.01, WT-IS vs. WT-IR) during reperfusion (**Figure 1C**), which, in part, could explain the lack of effects of the inhibitor on post-ischemic recovery of cardiac contractility. Additionally, SIRT3^-/-^ hearts demonstrated more cell damage during IR since LDH activity in the cardiac effluent released from SIRT3^-/-^ hearts at reperfusion was a 2.4-fold (*P* < 0.001) higher than that from WT hearts. Interestingly, SfA-treated SIRT3^-/-^ hearts exhibited reduced LDH release during the first 10 min of reperfusion (**Figure 1D**, S3-IR vs. S3-IS). In conclusion, these data suggest that SIRT3 is essential for maintenance of cardiac function after IR and reducing cellular damage.

### SIRT3^-/-^ Mice have High SIRT4 Expression in Cardiac Mitochondria

**FIGURE 2:**
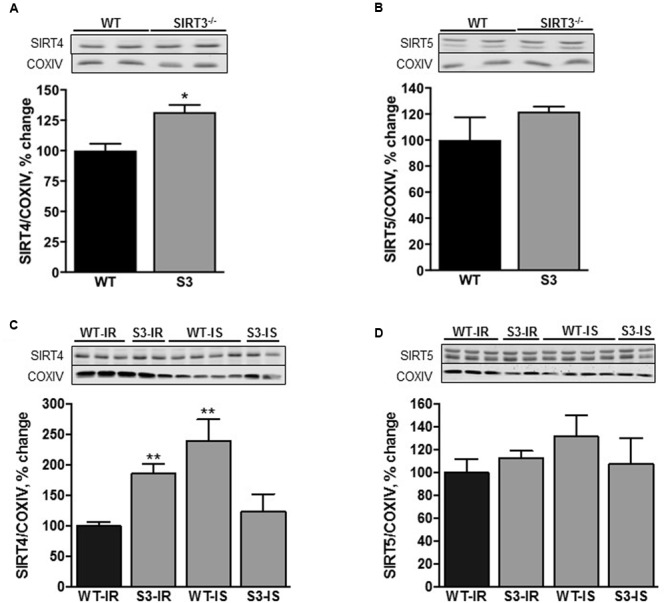
**SIRT4 (A,C)** and SIRT5 **(B,D)** levels in cardiac mitochondria isolated from WT and SIRT3^-/-^ non-perfused **(A,B)** or perfused **(C,D)** mice. Representative immunoblots (*upper panels*, **A**–**D**) of the proteins were obtained by Western blot analysis. Proteins were normalized to cytochrome c oxidase (COXIV), a mitochondrial housekeeping protein, and expressed as percentage change relative to the WT group (*bottom panels*, **A–D**). Groups are the same shown in **Figure 1**. ^∗^*P <* 0.01, ^∗∗^*P <* 0.001 vs. WT.

Three sirtuin isoforms, SIRT3, SIRT4, and SIRT5 are present in the mitochondria and SIRT3 is the major mitochondrial deacetylase. We assessed if SIRT3 deletion had any effect on protein expression of SIRT4 and SIRT5 in cardiac mitochondria. As shown in **Figure 2A**, protein levels of SIRT4 were 32% (*P <* 0.05) higher in intact (non-perfused) mitochondria isolated from SIRT3^-/-^ hearts. However, SIRT3 ablation had no effect on SIRT5 expression (**Figure 2B**). It should be noted that we observed similar changes in liver mitochondria of SIRT3^-/-^ mice (Supplementary Figure S1A).

Interestingly, SIRT4 expression was 85% (*P* < 0.001) higher in SIRT3 deficient animals subjected to IR when compared to their WT counterparts (**Figure 2C**). Protein expression of SIRT5 had an increasing trend in SIRT3^-/-^ hearts with or without IR, however, the difference did not reach the conventional significance compared to WT hearts (**Figures 2B,D**). Interestingly, SfA stimulated SIRT4 expression by 140% (*P* < 0.01) in WT hearts exposed to IR with no effect on SIRT5 (**Figures 2C,D**). Similar results on the expression of SIRT4 and SIRT5 were observed in intact (no IR) liver mitochondria of WT and SIRT3^-/-^ mice (Supplementary Figure S1B). Altogether, these data demonstrate that SIRT3 ablation results in an increase in SIRT4 expression.

### Heart Mitochondria of SIRT3^-/-^ Mice are More Sensitive to Ca^2+^-Induced Swelling

**FIGURE 3:**
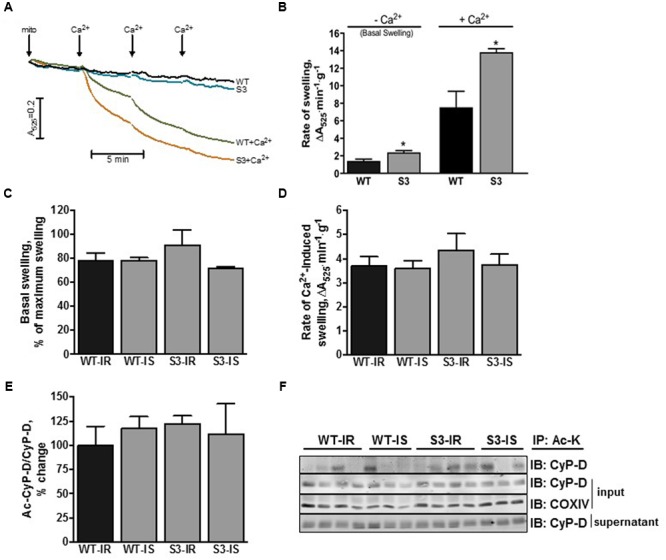
**Mitochondrial PTP opening and CyP-D acetylation in WT and SIRT3^-/-^ mice. (A)** Representative graph of mitochondrial swelling on intact WT and SIRT3^-/-^ cardiac mitochondria. Basal (non Ca^2+^-induced) mitochondrial swelling of **(B)** intact or **(C)** perfused WT and SIRT3^-/-^ hearts. Rate of mitochondrial Ca^2+^-induced swelling as a marker of mPTP opening of **(B)** intact or **(D)** perfused WT and SIRT3^-/-^ hearts measured as the slope at 200 mM of CaCl_2_. **(E)** Quantitative data of acetyl-CyP-D in mitochondria presented in **(F)**. **(F)** Representative immunoblots of acetyl-CyP-D in mitochondria isolated from hearts after IR. Acetylated proteins in liver and cardiac mitochondria were immunoprecipitated using antibodies against acetylated lysine, and then resolved by SDS-PAGE and immunoblotted using CyP-D antibodies. Groups are the same shown in **Figure 1**. S3, SIRT3^-/-^ mPTP formation from mitochondria of intact hearts in the absence of Ca^2+^; S3+Ca^2+^, SIRT3^-/-^ mPTP formation from mitochondria of intact hearts in the presence of Ca^2+^; WT, mPTP formation from mitochondria of WT intact hearta in the absence of Ca^2+^; WT+Ca^2+^, mPTP formation from mitochondria of WT intact hearts in the presence of Ca^2+^. ^∗^*P* < 0.05 vs. WT. *n* = 4–7 per group.

Next, we assessed mPTP opening in cardiac mitochondria isolated from WT and SIRT3^-/-^ mice with and without reperfusion. Mitochondria from intact SIRT3^-/-^ hearts displayed a 68% (*P* < 0.05) increase in their basal (non-Ca^2+^-induced) swelling as a marker of mPTP opening, compared to WT hearts (**Figures 3A,B**). *Addition of Ca^2+^ to* SIRT3 deficient mitochondria induced an 84% (*P* < 0.05) increase in the rate of mitochondrial swelling. These data suggest that SIRT3 plays an important role in preserving mitochondrial integrity under basal conditions and its downregulation leads to matrix swelling, which is further enhanced in the presence of Ca^2+^ (**Figures 3A,B**).

In contrast to intact hearts, SIRT3^-/-^ and WT hearts subjected to IR demonstrated similar basal (without added Ca^2+^) and Ca^2+^-induced swelling. Furthermore, SfA had no effect on mitochondrial swelling in SIRT3^-/-^ and WT hearts (**Figures 3C,D**). Analysis of CyP-D acetylation revealed no differences in acetyl-CyP-D levels in cardiac mitochondria between WT and SIRT3^-/-^ mice by the end of IR (**Figures 3E,F**). These data demonstrate that SIRT3^-/-^ mitochondria are more sensitive to Ca^2+^-induced mPTP induction under basal conditions, however, IR-induced oxidative stress equalizes the extent of mPTP opening and CyP-D acetylation in WT and SIRT3^-/-^ hearts.

**FIGURE 4:**
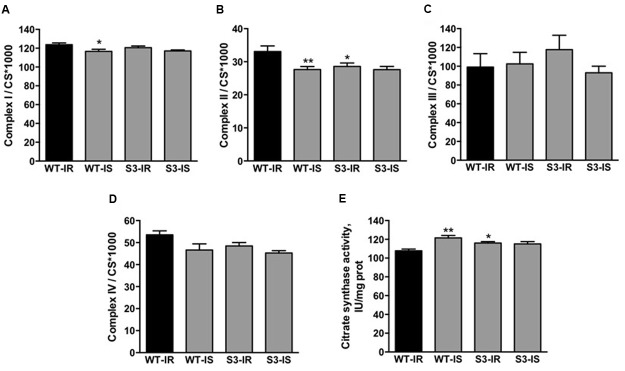
**Activity of ETC complexes I–IV (A–D)** and citrate synthase **(E)** in cardiac mitochondria of WT and SIRT3^-/-^ mice. Enzymatic activity of ETC complexes was measured spectrophotometrically using a specific substrate for each complex (see Materials and Methods). The activity of complexes was normalized to citrate synthase (CS) whereas CS activity was normalized to mg of mitochondrial protein. Groups are the same shown in **Figure 1**. ^∗^*P* < 0.05, ^∗∗^*P* < 0.01 vs. WT-IR. *n* = 5–7 per group.

SIRT3 has been shown to modulate the activity of ETC complexes through acetylation/deacetylation and therefore, we determined the enzymatic activity of ETC complexes in mitochondria isolated from WT and SIRT3^-/-^ hearts at the end of IR (**Figure 4A**). SIRT3^-/-^ hearts subjected to IR had similar activity to WT hearts for all ETC complexes, except complex II, which displayed a 14% (*P* < 0.05) decrease in activity compared to WT mitochondria (**Figure 4B**). The activity of citrate synthase was measured to examine the effect of SIRT3 ablation on mitochondrial mass (**Figures 4C,D**). The citrate synthase activity in SIRT3^-/-^ hearts was 8% (*P* < 0.05) higher than in WT hearts at the end of reperfusion (**Figure 4E**). In addition, SfA-treated WT hearts demonstrated a 13% (*P* < 0.001) increase in citrate synthase activity compared to the untreated WT hearts. Taken together, these data show that SIRT3 deficiency decreases complex II activity and increases mitochondrial mass.

### SIRT3^-/-^ Hearts Exhibit Increased Mitochondrial ROS Production after IR Injury

**FIGURE 5:**
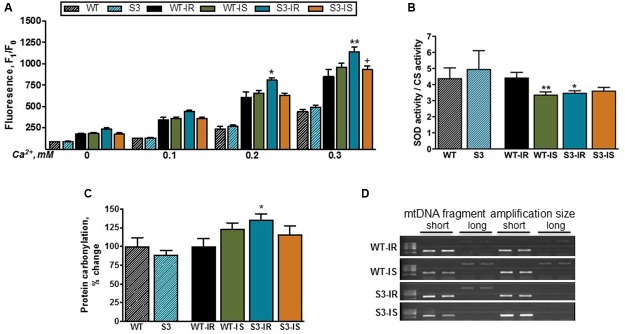
**Basal and Ca^2+^-stimulated ROS production (A)**, total SOD activity **(B)**, protein oxidation **(C)**, and mtDNA lesions **(D)** in mitochondria of WT and SIRT3^-/-^ hearts. ROS production **(A)** was assessed by measurement of resorufin fluorescence, the Amplex Red oxidation product. ROS production was stimulated by the repeated addition of Ca^2+^ to mitochondrial suspension. Total SOD activity **(B)** was normalized to the CS activity. Protein oxidation **(C)** was measured by dinitrophenylhydrazine derivatization to detect protein carbonyls in mitochondria, and normalized to the WT group (for S3) or WT-IR group (WT-IS, S3-IR, and S3-IS). mtDNA damage **(D)** was assessed by relative amplification of a long (15 kbp) to a short (2 kpb) DNA fragment (Damage to mtDNA reduces amplification of the long fragment). Groups are given in Section “Materials and Methods” (see *Ex vivo* Model of IR). ^∗^*P* < 0.05, ^∗∗^*P* < 0.001 vs. WT-IR; ^+^*P <* 0.05 ^++^*P <* 0.01 vs. S3-IR. *n* = 4–7 per group.

High ROS levels have been shown to play an essential role in mPTP formation, and SIRT3 has been suggested to increase the activity of SOD2 (mitochondrial SOD) through its deacetylation thereby preventing ROS accumulation. First, we determined ROS levels in mitochondria isolated from intact non-perfused WT and SIRT3^-/-^ hearts. As shown in **Figure 5A**, basal (no added Ca^2+^) and Ca^2+^-stimulated mitochondrial ROS production was not affected in WT and SIRT3^-/-^ hearts. Mitochondria isolated from intact WT and SIRT3^-/-^ hearts showed no difference in the enzymatic activity of SOD that represents both SOD1 in the intermembrane space and SOD2 in the matrix (**Figure 5B**).

We also assessed the protein carbonylation in cardiac mitochondria by derivatization, using DNPH, to detect protein carbonyls as a marker of protein oxidation. Like ROS production, SIRT3 ablation had no effect on the level of carbonylated proteins in mitochondria (**Figure 5C**). Similar data were obtained from intact liver mitochondria (Supplementary Figures S2A,B). However, IR had different effects on mitochondrial ROS production in WT and SIRT3^-/-^ hearts. Particularly, Ca^2+^ at concentrations of 0.2 and 0.3 mM increased mitochondrial ROS levels after IR in SIRT3^-/-^ hearts by 33% (*P* < 0.05) and 34% (*P* < 0.001), respectively, compared to WT hearts (**Figure 5A**). SfA reduced Ca^2+^-induced mitochondrial ROS production in SIRT3^-/-^ mice by 16% (*P* < 0.01) (**Figure 5A**). Mitochondrial SOD activity on SIRT3^-/-^ deficient hearts was 21% (*P* < 0.01) lower compared to perfused WT hearts after IR (**Figure 5B**, WT-IR vs. S3-IR).

Based on aforementioned data on ROS production and SOD activity, we examined next whether increased ROS production had any detrimental effects on mitochondrial proteins and DNA. As shown in **Figure 5C**, IR induced a 36% (*P* < 0.05) increase in carbonylation of total mitochondrial proteins in SIRT3 deficient hearts confirming the potential role of ROS in oxidative damage of proteins. In addition, we examined the presence of mtDNA lesions in WT and SIRT3^-/-^ hearts. Increased ROS can induce DNA damage through oxidation of guanine bases to 8-oxo-dG, which results in a DNA lesion causing a point mutation. However, we found no difference in mtDNA damage between WT and SIRT3^-/-^ hearts (**Figure 5D**) suggesting that mtDNA was not affected by SIRT3 ablation (Supplementary Figures S3A,B). Similar results were obtained in mitochondria isolated from the intact liver of WT and SIRT3^-/-^ mice (Supplementary Figures S3C,D).

In conclusion, under physiological conditions we found no difference in mitochondrial ROS production and protein carbonylation between WT and SIRT3^-/-^ hearts. However, in response to IR-induced oxidative stress, SIRT3 ablation enhanced ROS production and reduced SOD activity in mitochondria leading to increased oxidative damage to mitochondrial proteins.

## Discussion

This study demonstrates that SIRT3 ablation does not induce an increase in ROS production or protein oxidation in cardiac mitochondria presumably due to activation of compensatory mechanisms such as upregulation of SIRT4. However, exposure of SIRT3^-/-^ hearts to IR diminishes post-ischemic recovery of cardiac function associated with increased ROS production and protein oxidation (**Figures 1A,B**, **5A,C**). Inhibition of the mPTP does not improve recovery of cardiac function after ischemia in both WT and SIRT3^-/-^ hearts although it significantly reduces ROS production and LDH release in the coronary effluent from SIRT3^-/-^ mice. Interestingly, this study demonstrates for the first time that SIRT3 deficient mitochondria are more sensitive to Ca^2+^-induced mPTP formation that could account for the significant reduction in post-ischemic recovery, suggesting an essential role for SIRT3 in mPTP-mediated IR cardioprotection.

The role of acetylation in the pathogenesis of cardiovascular and neurodegenerative diseases, diabetes, cancer, and aging is broadly discussed. The activity of the main mitochondrial proteins involved in mitochondrial metabolism and ATP synthesis is regulated through acetylation/deacetylation predominantly by SIRT3 (Hirschey et al., 2010; Yu et al., 2012; Vassilopoulos et al., 2014). Overexpression of SIRT3 abrogated triptolide-induced cell death in H9c2 cardioblasts (Yang et al., 2016), doxorubicin-induced cardiomyocyte death, hypertrophy, ROS production, and mtDNA damage (Pillai et al., 2016), and protected rat cardiomyocytes against hypertrophy and ROS overproduction (Sundaresan et al., 2009). Conversely, downregulation or absence of SIRT3 was associated with decreased cardiac recovery and increased infarct size (Porter et al., 2014), enhanced doxorubicin-induced cardiac hypertrophy (Pillai et al., 2016) and H_2_O_2_-induced neuronal injury (Dai et al., 2014). SIRT3 has also been implicated in regulation of mitochondrial antioxidant system.

Cardiac mitochondria of SIRT3^-/-^ mice exhibited increased expression of SIRT4 suggesting the development of a possible compensatory mechanism (**Figure 2A**). This conclusion was supported by our findings in liver mitochondria of SIRT3^-/-^mice that also displayed high SIRT4 expression with no change in mitochondrial protein acetylation (Supplementary Figures S1A,C). Indeed, most recent data demonstrated that SIRT4 overexpression significantly attenuated reduced ROS production and attenuated apoptosis in response to inflammation-induced oxidative stress (Shi et al., 2017). Interestingly, previous studies (Porter et al., 2014; Koentges et al., 2016) and our data suggest that the compensatory mechanisms developed in SIRT3^-/-^ mice protect the heart against mild but not severe ischemia. No difference was found in post-ischemic recovery between WT and SIRT3^-/-^ hearts after mild ischemia (Koentges et al., 2016). No effects of IR can be associated with shortness (17.5 min) of the ischemic period, which may have mild oxidative stress on WT and SIRT3^-/-^ hearts (Supplementary Figures S2C–F). It should be noted that the *in vivo* coronary artery ligation technique is different from the *ex vivo* global IR model used in our experiments. Also, differences in preparation of the hearts for *in vivo* surgery and *ex vivo* Langendorff-mode perfusion may have divergent effects on post-ischemic recovery. Koentges et al. (2016) subjected the hearts to a brief period of hypothermia while we cannulated the aorta *in vivo* and then, quickly excised and perfused it *ex vivo*, a process that takes no longer than 20 s. Additionally, isoflurane anesthesia used in *in vivo* studies (Koentges et al., 2016) may have cardioprotective effects on WT and SIRT3^-/-^ hearts. However, the studies with the same ex-vivo global ischemia model used in our studies demonstrated that the hearts of heterozygous mutant (SIRT3^-/+^) mice were more sensitive to severe IR injury (Porter et al., 2014). Likewise, our data show that complete SIRT3 ablation during severe IR injury decreased post-ischemic recovery of cardiac work (RPP) in these hearts (**Figure 1B**).

Previous studies have demonstrated that increased acetylation of mitochondrial proteins in the heart in response to oxidative stress, such as IR, is associated with mPTP opening and acetylation of CyP-D (Hafner et al., 2010; Parodi-Rullan et al., 2012). Indeed, SIRT3 deficiency enhanced basal and Ca^2+^overload-induced mitochondrial swelling although a direct cause-and-effect relationship remains to be elucidated. We found no difference in mPTP opening between WT and SIRT3^-/-^ hearts subjected to IR that can be explained by the maximum swelling state and increased cell death that was reached in both WT and SIRT3^-/-^ mitochondria by the end of IR. Likewise, no differences in acetylated CyP-D levels might be due to a maximum effect of IR which equalizes acetylation levels of CyP-D by the end of reperfusion in WT and SIRT3^-/-^ mitochondria. Since sirtuins are NAD^+^ dependent deacetylases, low NAD^+^ levels during reperfusion could lead to decreased SIRT3 activity and global mitochondrial protein acetylation, including CyP-D, in WT animals. However, further studies need to establish the cause-and-effect relationship between CyP-D acetylation and mPTP opening in response to oxidative stress in SIRT3^-/-^ hearts. In addition to acetylation, other unknown mechanisms may be involved in CyP-D activation in SIRT3 deficient mitochondria. Upon activation, CyP-D can induce mPTP opening through binding to ANT (Woodfield et al., 1998), P_i_C (Leung et al., 2008), and F_0_F_1_-ATP synthase (Giorgio et al., 2009) in the inner mitochondrial membrane. Additionally, it can regulate the mPTP through interactions with proteins such as p53 (Vaseva et al., 2012), and PPARα (Barreto-Torres et al., 2015; Barreto-Torres and Javadov, 2016) in the matrix.

Excessive oxidative stress such as severe IR leads to mitochondrial ROS accumulation due to imbalance between ROS production and antioxidant capacity. High ROS along with increased Ca^2+^ and P_i_ are the contributing factors for mPTP induction. The effect of protein acetylation, particularly CyP-D acetylation, on ROS production and mPTP opening remains unclear. SIRT3 deficiency had no additional effects on mitochondrial H_2_O_2_ production, protein carbonylation, and DNA lesions in intact hearts and liver. However, IR injury significantly stimulated Ca^2+^-induced mitochondrial ROS production, and reduced antioxidant capacity of mitochondria, particularly SOD activity associated with increased protein oxidation in SIRT3^-/-^ hearts. These results suggest that, under physiological conditions, SIRT3 ablation has detrimental effects on mitochondrial function that cannot be seen due to the possible development of compensatory mechanisms to overcome these alterations such as SIRT4 overexpression. However, IR allows the unmasking of mitochondrial and cardiac dysfunction caused by SIRT3 deficiency resulting in high vulnerability of SIRT3^-/-^ hearts to oxidative stress.

The immunosuppressive drugs CsA and SfA are well-known inhibitors of CyP-D that exert cardioprotection against IR in various animal models (Reviewed in Hausenloy et al., 2003; Javadov et al., 2009; Halestrap and Richardson, 2015). Interestingly, SfA had no effect on post-ischemic recovery or mPTP in WT and SIRT3^-/-^ hearts. CsA and SfA work in a narrow concentration range (Griffiths and Halestrap, 1993) and, although previous studies have shown a cardioprotective potential for SfA in rats (Clarke et al., 2002), this is the first study using SfA in an *ex vivo* model of IR in mice. Furthermore, a significant difference in sensitivity between species (rats vs. mice) to CyP-D inhibition could also account for the observed effects. Severe stress present during IR could overpower the mPTP inhibitory effects of SfA as it is known to increase the threshold for mPTP formation but does not prevent mPTP at high Ca^2+^ concentrations. Also, SfA-induced vasoconstriction of coronary arteries as evident by increased coronary pressure in WT animals as well as timing of SfA administration could account for the lack of protective effects. SfA was shown to decrease infarct size when administered during early (no later than first 15 min) reperfusion (Hausenloy et al., 2003). In addition, chronic inhibition of CyP-D can diminish physiological function of CyP-D and low-conductance mPTP opening in the cell (Elrod et al., 2010). SIRT3 deacetylates CyP-D on K166, adjacent to the binding site of CsA (Hafner et al., 2010), although the binding site and inhibitory mechanism for SfA is different from that for CsA. We suggested that either acetylation may mask the binding site and prevent inhibitory effects of SfA or a high affinity SfA binding to acetylated CyP-D can exert beneficial effects in the SIRT3^-/-^ hearts subjected to IR. Indeed, we are the first to attempt SfA administration to SIRT3 deficient animals where acetylation could impede SfA binding to CyP-D, although more in depth studies need to be done to establish this relationship. However, SfA reduced LDH release and prevented protein carbonylation, and ROS production associated with restoration of SOD2 activity in SIRT3^-/-^ hearts.

Despite reduced post-ischemic recovery, SIRT3^-/-^ hearts subjected to IR demonstrated similar to WT hearts activity for all ETC complexes, except complex II. The effects of IR on the enzymatic activity of ETC complexes are widely debated (Lesnefsky et al., 2001) and can be different depending on the severity of IR, animal species, techniques for enzymatic assay, normalization of results (per mg mitochondrial protein or citrate synthase activity), etc. Alterations in the activity of ETC complexes were demonstrated in mitochondria isolated from perfused rabbit (Chen et al., 2010) and guinea pig (Gadicherla et al., 2012) hearts after IR injury. On the other hand, studies from our (Jang et al., 2016) and others’ groups (Tompkins et al., 2006) demonstrated that sustained IR can induce mitochondrial dysfunction without significant changes in the enzymatic activity of ETC complexes. Most likely, respiratory rates of mitochondria can be compromised by IR-induced Ca^2+^ overload, high ROS, membrane depolarization and other alterations. Enzymatic activity of ETC complexes can be affected differently depending, among others, on the severity of oxidative stress.

## Conclusion

We found high vulnerability of SIRT3^-/-^ hearts to IR injury that demonstrated low post-ischemic recovery and increased mitochondrial ROS production and mitochondrial protein oxidation. Low post-ischemic recovery of SIRT3^-/-^ hearts was not associated with increased CyP-D acetylation. This might be due to increased acetylation of mitochondrial proteins including CyP-D by IR in WT hearts. At the same time, mitochondria of intact SIRT3^-/-^ hearts demonstrated an increase in Ca^2+^-induced mPTP formation suggesting a possible regulatory role for SIRT3 in mPTP induction. We observed no difference on ROS production and protein oxidation between intact WT and SIRT3^-/-^ hearts probably due to the development of compensatory mechanisms that result in the masking of dysregulated cellular processes. For instance, increased expression of SIRT4, a mitochondrial sirtuin, which reduces apoptosis induced by hypoxia-reoxygenation in H9c2 cells (Liu et al., 2013) can be involved in the compensatory mechanisms in SIRT3^-/-^ mice. However, exposure of hearts to oxidative stress induced by IR revealed significant differences between WT and SIRT3^-/-^ suggesting that adaptation is stress-load dependent. Indeed, a 50% decrease in ATP synthesis in SIRT3^-/-^ mice accounts for the maladaptation seen only during stressful conditions (Ahn et al., 2008). Overall, these studies demonstrate an important role of SIRT3 in post-ischemic recovery of cardiac function, inhibition of mPTP-mediated mitochondrial swelling, and ROS production in the heart.

### Study Limitations

One of the limitations of this study is the lack of data corroborating acetylation of CyP-D in mitochondria from SIRT3 deficient hearts. Due to a low yield of mitochondria (0.5–0.7 mg) from the heart of WT and SIRT3^-/-^ mice we were not able to assess CyP-D acetylation by the immunoprecipitation technique which requires a large amount of mitochondrial protein. Instead, we determined CyP-D acetylation in liver mitochondria isolated from SIRT3^-/-^ mice although they are metabolically different from cardiac mitochondria. In addition, WT and SIRT3^-/-^ hearts in the control group were rinsed in perfusion buffer, but not perfused. Although the effects of normal perfusion on the heart is negligible and can be ignored, comparison of non-perfused hearts with perfused hearts is considered a limitation of the study.

## Author Contributions

RP-R, XC-D, PR, and SeJ performed experiments and analyzed data. RP contributed to the interpretation of data and prepared a draft of the paper. SaJ designed the study, interpreted the data, and critically revised the paper. All authors participated in the interpretation of data, reviewed and approved the final version of the paper.

## Conflict of Interest Statement

The authors declare that the research was conducted in the absence of any commercial or financial relationships that could be construed as a potential conflict of interest.

## Abbreviations

APE-1: apurinic/apyrimidinic endonuclease
CyP-D: cyclophilin D
CsA: cyclosporine A
ETC: electron transport chain
IR: ischemia-reperfusion
KO: knockout
LVDP: left ventricular developed pressure
LVEDP: left ventricular end diastolic pressure
LVSP: left ventricular systolic pressure
mtDNA: mitochondrial DNA
mPTP: mitochondrial permeability transition pore
OGG1: 8-oxoguanine DNA glycosylase
ROS: reactive oxygen species
RPP: rate pressure product
SfA: sanglifehrin A
SIRT3^-/-^: SIRT3 KO
SOD: superoxide dismutase
WT: wild type.
