# Corrigendum: High Sensitivity of SIRT3 Deficient Hearts to Ischemia-Reperfusion Is Associated with Mitochondrial Abnormalities

**DOI:** 10.3389/fphar.2017.00439

**Published:** 2017-06-28

**Authors:** Rebecca M. Parodi-Rullán, Xavier Chapa-Dubocq, Pedro J. Rullán, Sehwan Jang, Sabzali Javadov

**Affiliations:** Department of Physiology, University of Puerto Rico School of MedicineSan Juan, PR, United States

**Keywords:** SIRT3, heart, ischemia-reperfusion, mitochondria, protein acetylation, sanglifehrin A

In the original article, due to random loading of the groups and their vertical arrangement, we presented individual lanes from the same gel arranged as a representative image in Figure S3D of the Supplementary Material (in contrast to Figure 5D, where the arrangement was horizontal and the samples are arranged according to the groups). Since this explanation was not given in Figure legends, an original section of the gel as a correct representative image for Figure S3D is shown below. The authors apologize for this error and state that this does not change the scientific conclusions of the article in any way.

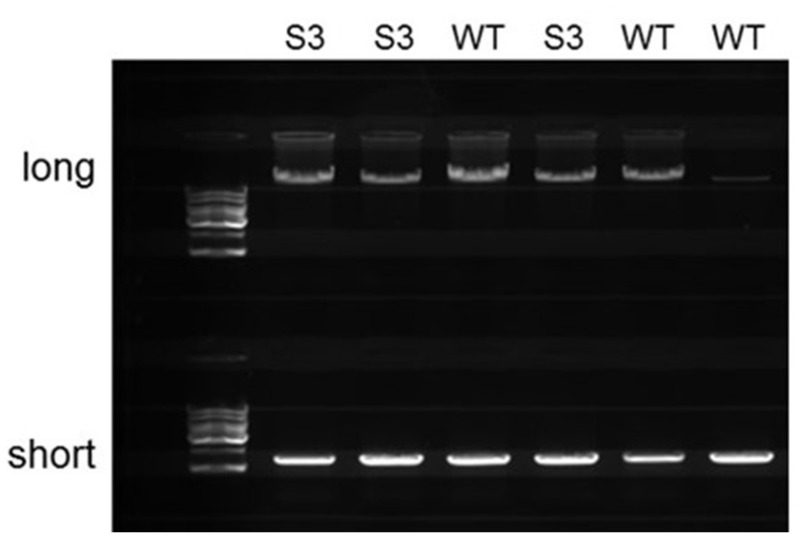



## Conflict of interest statement

The authors declare that the research was conducted in the absence of any commercial or financial relationships that could be construed as a potential conflict of interest.

