# Supplementary material for: High Sensitivity of SIRT3 Deficient Hearts to Ischemia-Reperfusion Is Associated with Mitochondrial Abnormalities
Source: Front Pharmacol. 2017 May 16;8:275. doi: 10.3389/fphar.2017.00275 (PMC5432544; doi:10.3389/fphar.2017.00275)
Supplement: Supplementary file 1 [file Data_Sheet_1.PDF]

SUPPLEMENTARY MATERIAL

Supplementary Figure Legends

Figure S1. SIRT4 (***A***), SIRT5 (***B***), and total lysine-acetylated protein (***C***) levels in liver mitochondria isolated from WT and SIRT3$^{-/-}$ mice. Representative immunoblots (*upper panels*, ***A***, ***B***, and ***C***) of the proteins were obtained by Western blot analysis. Proteins were normalized to cytochrome c oxidase (COXIV), a mitochondrial housekeeping protein, and expressed as percentage change relative to the WT group (*bottom panels*, ***A***, ***B***, and ***C***). *P<0.01, **P<0.001 vs. WT. n=7 per group.

Figure S2. Antioxidant capacity of liver mitochondria in WT and SIRT3$^{-/-}$ mice. (***A***) Protein oxidation measured by DNPH derivatization to detect protein carbonyls; (***B***) Total antioxidant capacity assessed by measuring the reduction of Cu^{2+} in Trolox equivalents; (***C***) Protein levels of SOD2 determined by SDS-PAGE and Western blotting using SOD2 antibodies; (***D***) Acetylation of SOD2 determined by immunoprecipitation of mitochondrial proteins with acetylated lysine antibodies followed by immunoblotting with antibodies against SOD2; (***E***) Enzymatic activity of mitochondrial SOD as percent inhibition rate of WST-1 formazan; (***F***) Percent inhibition rate of mitochondrial SOD normalized to SOD2 protein levels. *P<0.05 vs. WT. n=7-9 per group.

Figure S3. Protein expression of the base excision repair enzymes OGG1 (***A***) and APE-1 (***B***), total DNA 8-oxo-dG lesions (**C**), and mtDNA lesions (***D***) from WT and SIRT3$^{-/-}$ intact liver. Protein levels of OGG1 and APE-1 were analyzed after resolving of mitochondrial proteins by SDS-PAGE followed by immunoblotting with specific antibodies against OGG1 and APE-1. *P<0.01 vs. WT. n=7-10 per group.

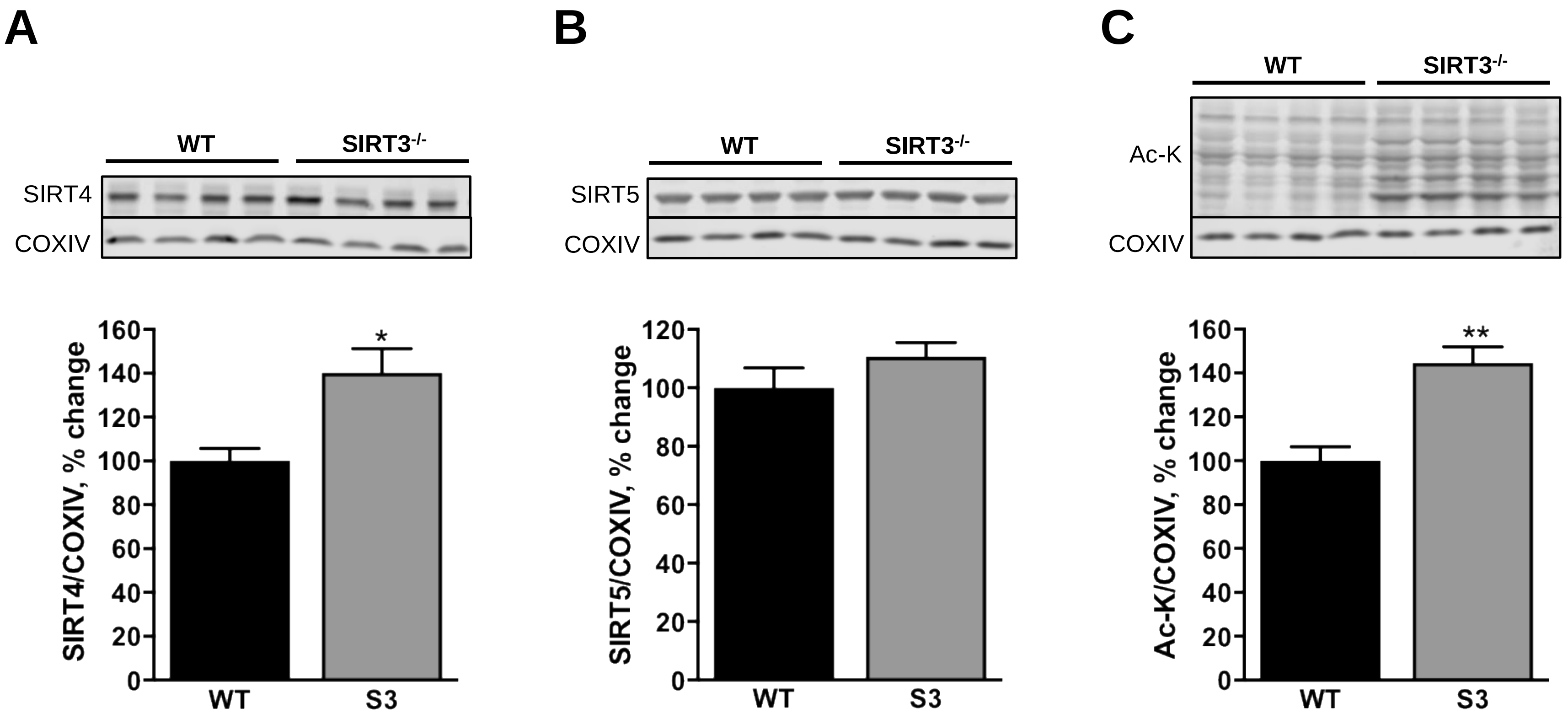
A
B
C
WT
SIRT3-/-
SIRT4
COXIV
SIRT5
Ac-K
SIRT4/COXIV, % change
SIRT5/COXIV, % change
Ac-K/COXIV, % change
0
20
40
60
80
100
120
140
160
*
**
WT
S3

Figure S1

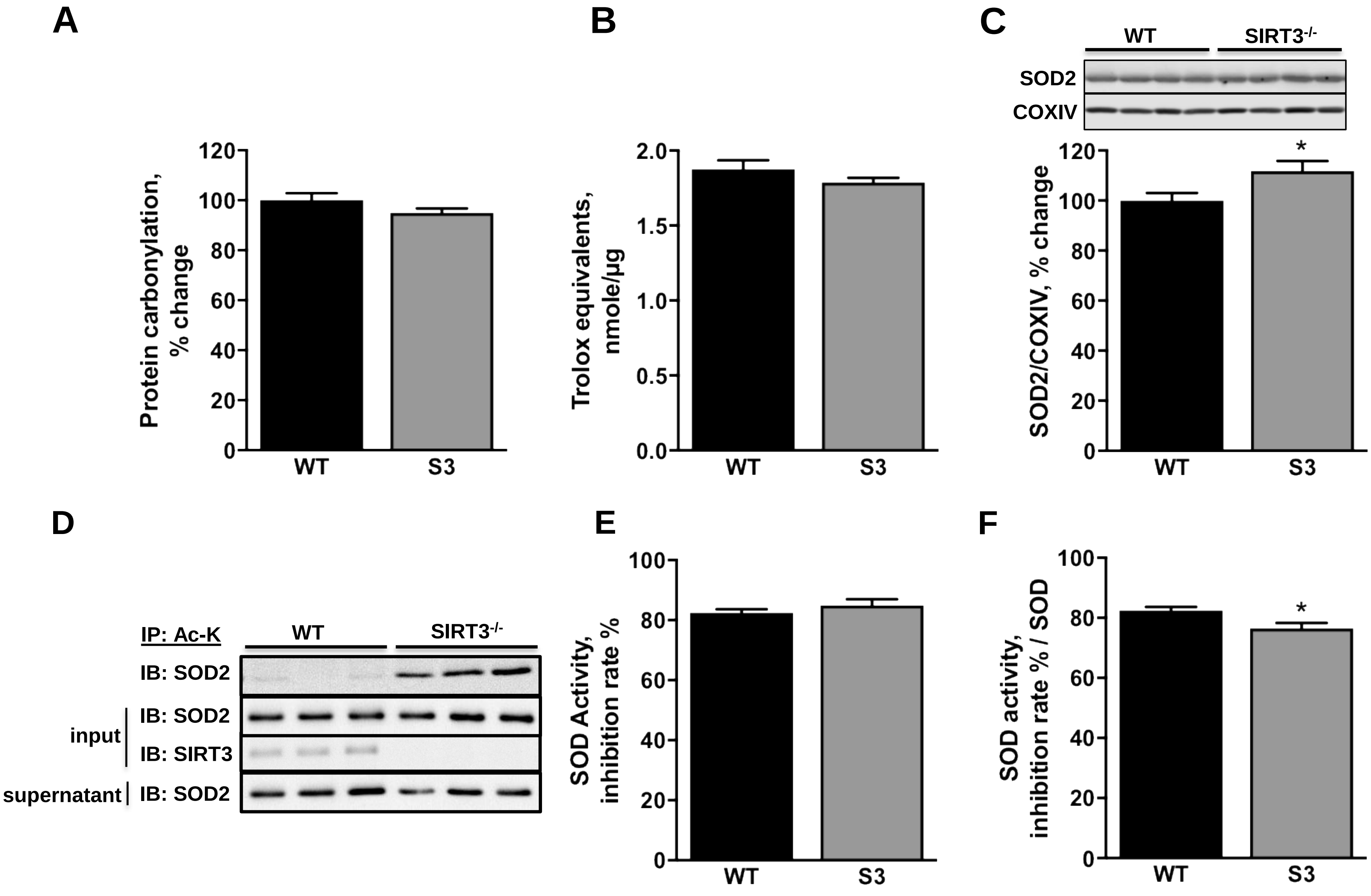
A
Protein carbonylation, % change
120
100
80
60
40
20
0
WT
S3
B
Trolox equivalents, nmole/µg
2.0
1.5
1.0
0.5
0.0
WT
S3
C
WT
SIRT3-/-
SOD2
COXIV
SOD2/COXIV, % change
120
100
80
60
40
20
0
*
WT
S3
D
IP: Ac-K
WT
SIRT3-/-
IB: SOD2
input
IB: SOD2
IB: SIRT3
supernatant
IB: SOD2
E
SOD Activity, inhibition rate %
100
80
60
40
20
0
WT
S3
F
SOD activity, inhibition rate % / SOD
100
80
60
40
20
0
*
WT
S3

Figure S2

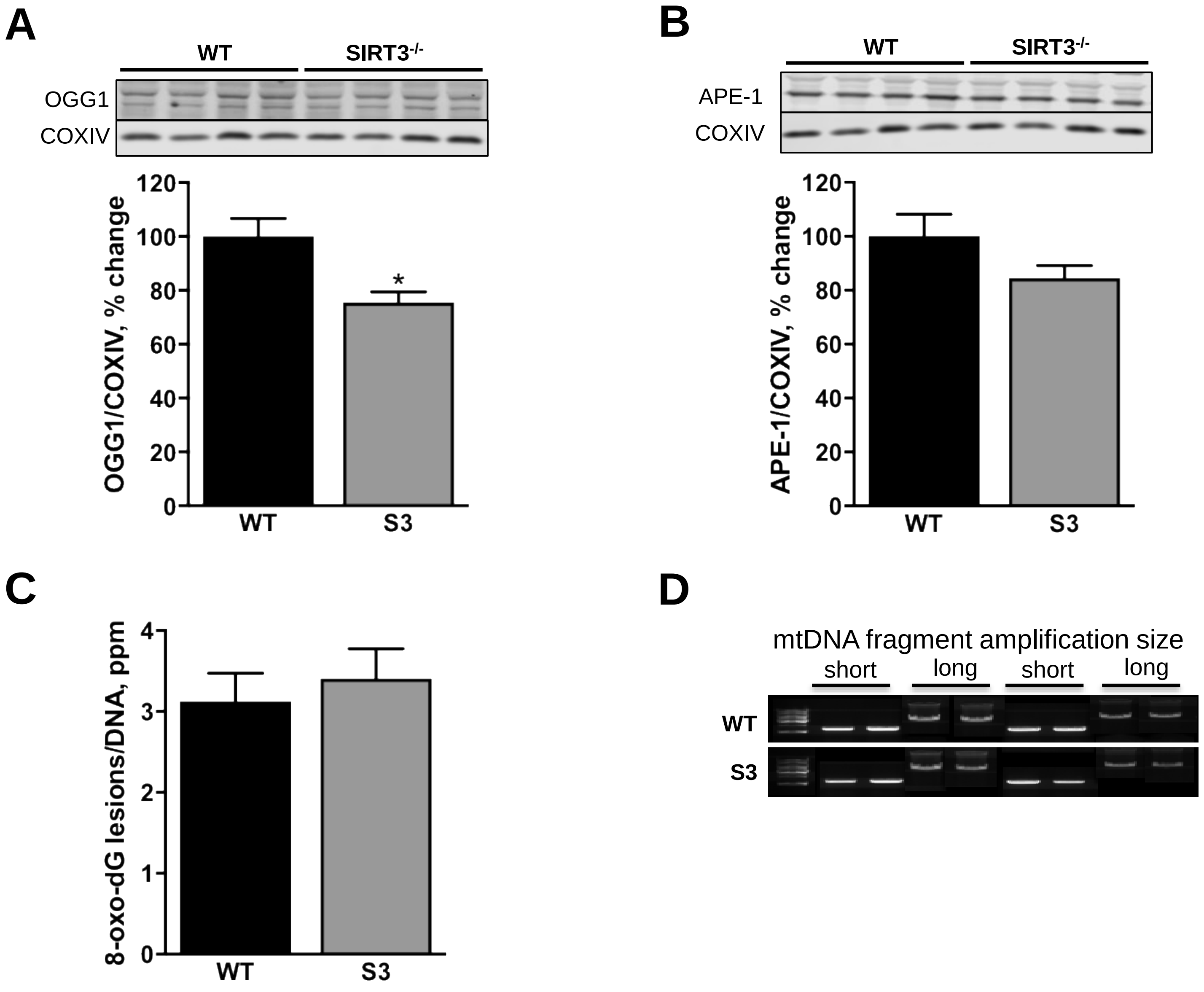
A
WT
SIRT3-/-
OGG1
COXIV
OGG1/COXIV, % change
120
100
80
60
40
20
0
*
WT
S3
B
WT
SIRT3-/-
APE-1
COXIV
APE-1/COXIV, % change
120
100
80
60
40
20
0
WT
S3
C
8-oxo-dG lesions/DNA, ppm
4
3
2
1
0
WT
S3
D
mtDNA fragment amplification size
short
long
short
long
WT
S3

Figure S3
